# Identification of potential modulators for human GPD1 by docking-based virtual screening, molecular dynamics simulations, binding free energy calculations, and DeLA-drug analysis

**DOI:** 10.1038/s41598-024-61439-y

**Published:** 2024-06-19

**Authors:** Anzheng Hu, Hongwei Chen, Wenwei Pang, Xiaojie Pu, Zhongquan Qi, Haiyan Chen

**Affiliations:** Guangxi Key Laboratory of Special Biomedicine, School of Medicine, Guangxi University, Nanning, 530004 Guangxi China

**Keywords:** GPD1, Virtual screening, Molecular docking, Molecular dynamics simulation, Binding free energy calculations, Virtual drug screening, Drug discovery

## Abstract

Cytosolic Glycerol-3-phosphate dehydrogenase 1 (GPD1, EC 1.1.1.8) plays a pivotal role in regulating the Embden-Meyerhof glucose glycolysis pathway (E-M pathway), as well as in conditions such as Huntington’s disease, cancer, and its potential role as a specific marker for Dormant Glioma Stem Cells. In this study, we conducted virtual screening using the ZINC database (http://zinc.docking.org/) and the GPD1 structure to identify potential GPD1 modulators. The investigation involved screening active candidate ligands using ADMET (Absorption, Distribution, Metabolism, Excretion, Toxicity) parameters, combined with molecular docking, pose analysis, and interaction analysis based on Lipinski and Veber criteria. Subsequently, the top 10 ligands were subjected to 200 ns all-atom molecular dynamics (M.D.) simulations, and binding free energies were calculated. The findings revealed that specific residues, namely TRP14, PRO94, LYS120, ASN151, THR264, ASP260, and GLN298, played a crucial role in ensuring system stability. Furthermore, through a comprehensive analysis involving molecular docking, molecular M.D., and DeLA-Drug, we identified 10 promising small molecules. These molecules represent potential lead compounds for developing effective therapeutics targeting GPD1-associated diseases, thereby contributing to a deeper understanding of GPD1-associated mechanisms. This study's significance lies in identifying key residues associated with GPD1 and discovering valuable small molecules, providing a foundation for further research and development.

## Introduction

**Figure 1:**
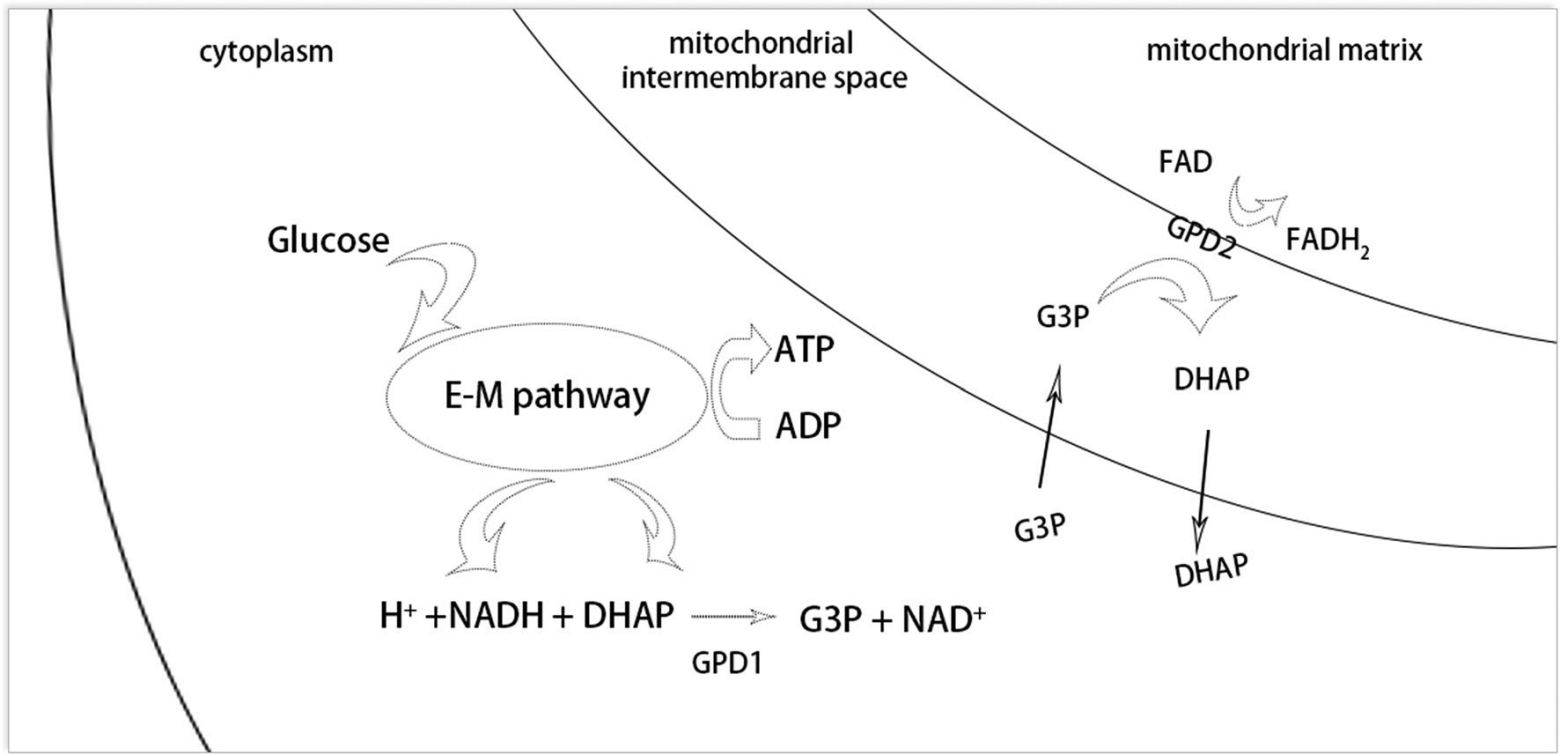
The reversible biochemical reactions catalyzed by GPD1 and GPD2 in the glycerol-3-phosphate shuttle.

Glycerol-3-phosphate dehydrogenase 1 (GPD1) is a crucial NAD+/NADH-dependent enzyme located in the cytosol, playing a pivotal role in the reversible conversion of glycerol-3-phosphate (G3P) to dihydroxyacetone phosphate (DHAP). Concurrently, within the Embden-Meyerhof (E-M) glycolysis pathway, DHAP and NADH are transformed into G3P and NAD^+^, respectively, facilitating further metabolic activities (Fig. 1). In contrast, mitochondrial glycerol-3-phosphate dehydrogenase 2 (GPD2, EC 1.1.5.3) serves as a flavin-dependent dehydrogenase, tightly bound to the outer surface of the inner mitochondrial membrane^1^. It catalyzes the irreversible oxidation of glycerol-3-phosphate to dihydroxyacetone phosphate, with Flavine Adenine Dinucleotide (FAD) being reduced to FADH_2_ (Fig. 1). The concerted actions of GPD1 and GPD2 establish the glycerol-3-phosphate shuttle, a mechanism enabling the reoxidation of NADH produced in the cytosol, primarily from glycolysis, to NAD^+^ by the mitochondrial electron-transport chain^2^. This shuttle is critical in transferring reducing equivalents from cytosolic NADH into the mitochondrial matrix^3,4^.

Therefore, GPD1 plays a multifaceted role in various metabolic activities, including its implication in obesity control and treatment^5^ and its involvement in the pathogenesis of Rheumatoid arthritis^6^. Notably, studies in yeast cells have illuminated the significant role of GPD1 in regulating mutant proteins, particularly in the context of Huntington’s disease. This neurodegenerative disorder, linked to the length and aggregation of polyglutamine channels in Huntington’s proteins, is deeply influenced by GPD1^7^.

Furthermore, GPD1's expression in brain tumor stem cells (BTSCs) establishes it as a specific marker for dormant and drug-resistant BTSCs. Targeting GPD1 has shown promise in disrupting BTSCs and prolonging their survival^8^. Concurrently, as stem cell exosomes gain prominence in therapeutic research^9,10^, the relationship between GPD1 and tumor development becomes increasingly evident^11,12^. GPD1 emerges as a regulator of stem cell exosome secretion, impacting various cancers such as renal clear cell carcinoma, bladder, prostate, breast, and epithelial ovarian cancer^13–16^. This association positions GPD1 as a valuable research object, suggesting its potential role in regulating stem cell exosome secretion. The findings of GPD1 studies underscore its high research value and potential significance in therapeutic interventions.

**Figure 2:**
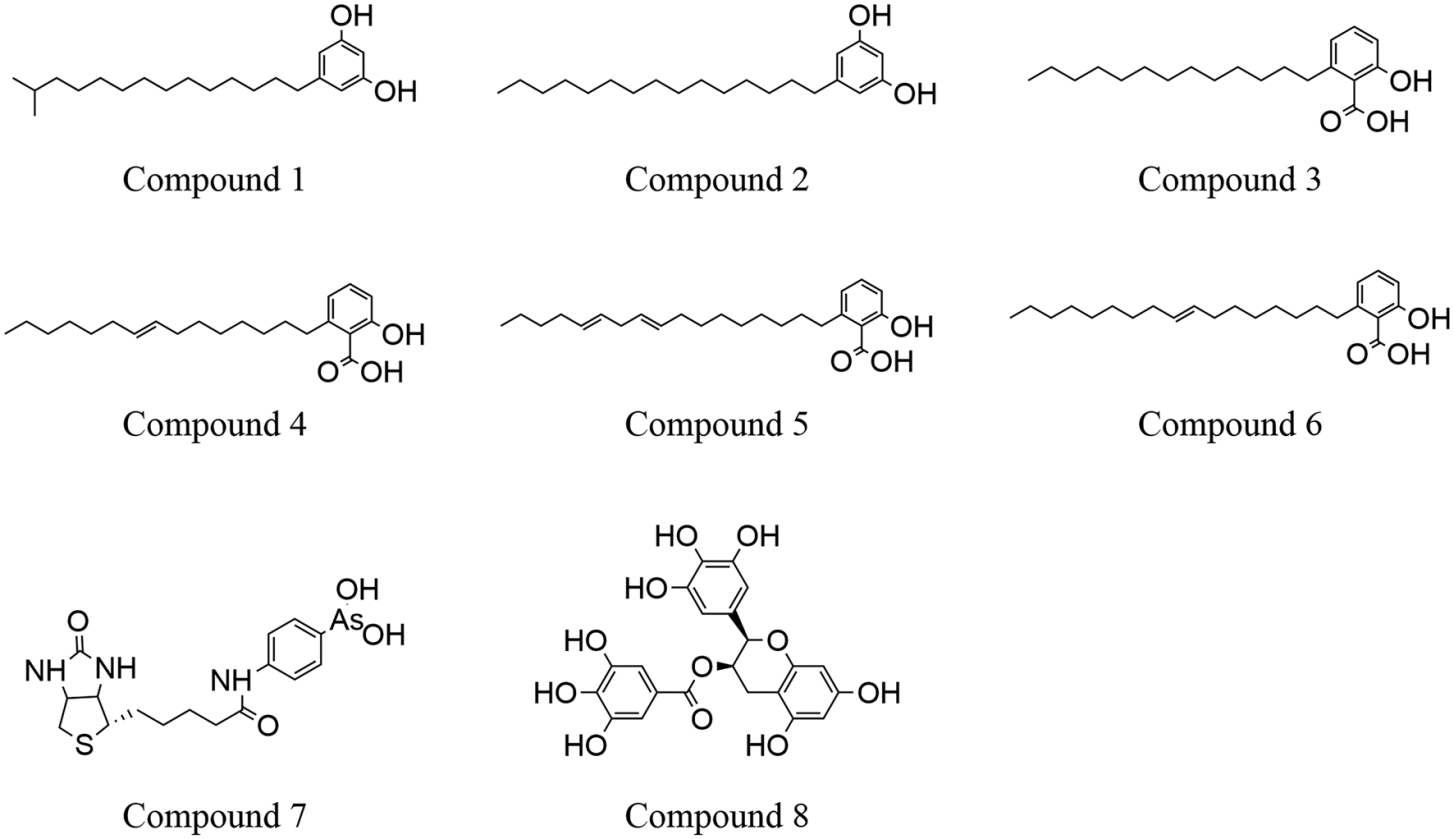
Glycerol-3-phosphate dehydrogenase 1 inhibitors as reported in the literature.

Several inhibitors of GPD1 have been reported (Fig. 2)^17–20^. Compound 1 and Compound 2^17^ demonstrated IC_50_ values of 4.1 μM and 4.5 μM, respectively. Compound 8^18^, identified in green tea catechins known for regulating fat and obesity accumulation, exhibited a 50% inhibition effect at a concentration of approximately 20 μM. Compounds 3, 4, 5, and 6^19^, isolated from *Ginkgo biloba,* displayed notable inhibitory effects, with reported IC_50_ values of 9.4, 4.0, 4.6, and 2.4 μg/ml, respectively. Furthermore, Compound 7^20^, a toxin, nearly completely inhibited GPD1 activity at 1 μM.

In this study, we employ a series of computer-assisted approaches to elucidate the binding mode of GPD1 protein with ligands, providing a more convenient and intuitive understanding. The goal is to explore novel lead compounds. Subsequently, utilizing DeLA-Drug, an artificial intelligence-based platform, we generate new compounds that maintain drug-likeness and synthetic accessibility. The results are then subjected to ADMET and molecular docking analyses, yielding a series of well-bound small molecules.

## Materials and methods

**Figure 3:**
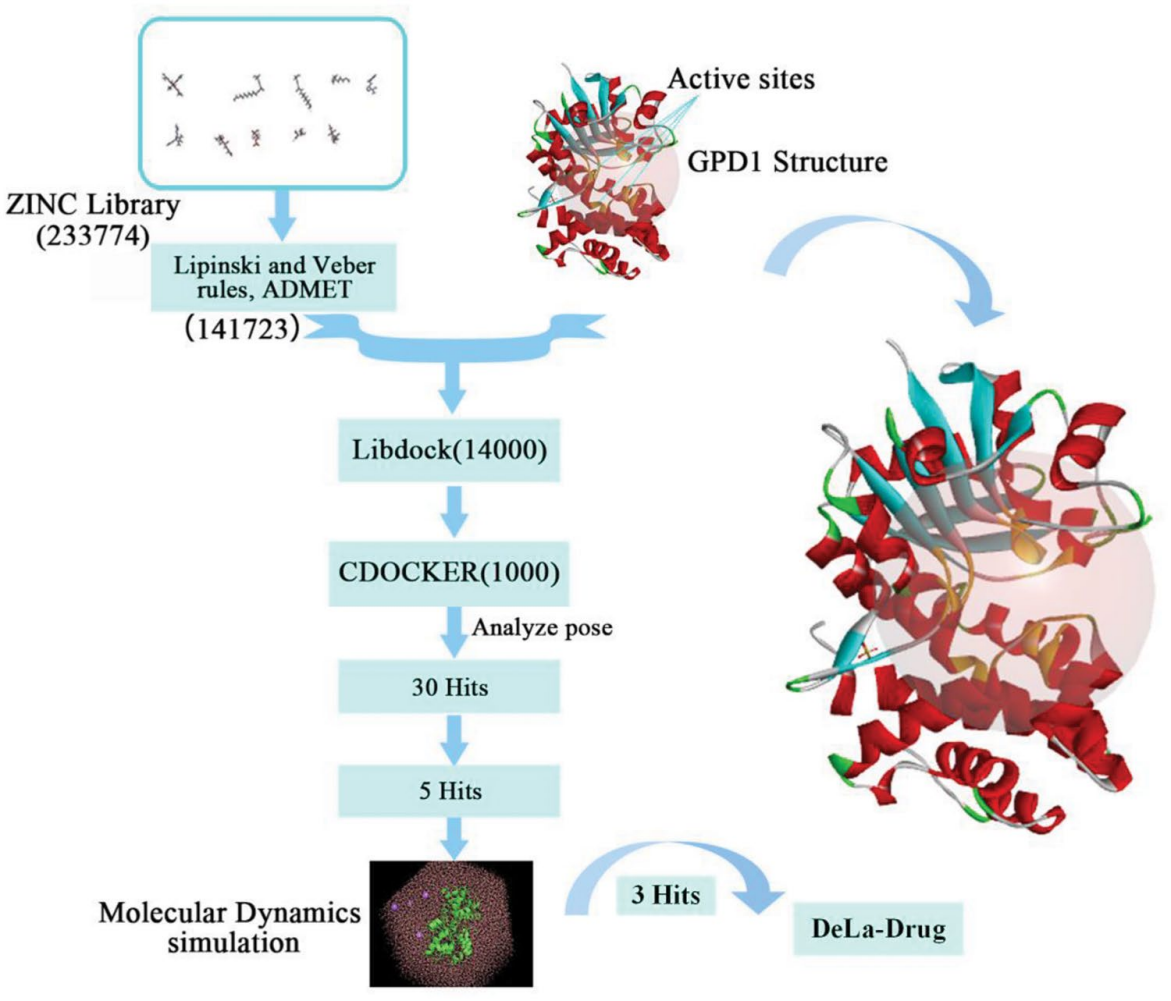
Virtual screening flowchart.

The research encompassed several key steps, including the downloading and processing of GPD1 (Supplementary Information), virtual screening, molecular docking, protein–ligand composition analysis, and subsequent molecular dynamics (M.D.) simulations (Fig. 3) and free energy calculations for five selected small molecules alongside one positive small molecule. The software tools utilized included Discovery Studio 2019 (D.S.) for protein and small molecule, docking, and analysis, while GROMACS was employed for simulating protein–ligand complexes. Additionally, DelaDrug, a freely accessible platform (http://www.ba.ic.cnr.it/softwareic/deladrugportal/), was employed to generate drug-like analogues.

### Preparation of protein and ligands

#### Preparation of protein

The GPD1 protein complex (PDB ID, 1WPQ) was obtained from the RCSB PDB website (https://www.rcsb.org/). Using D.S. software, the B chain of the protein was prepared by necessary cleaning, removal of water molecules, and energy minimization. Active sites (AC8, BC2) were marked in yellow at positions corresponding to the co-crystallized ligand, and these positions were chosen to construct the active pocket (Fig. 3).

#### Preparation of small molecules

The ZINC15 database (http://zinc.docking.org/) provided a collection of 233,774 3D small molecules classified as Lead-like and in-stock. The initial step involved downloading this database to facilitate subsequent molecular docking procedures. The preparation work for molecular docking was executed using Discovery Studio (D.S.) software. First, the downloaded small molecule sets were subjected to a screening process employing Lipinski and Veber rules. Following this screening, the selected small molecule sets underwent further refinement and analysis utilizing the ADMET tool.

To enhance the quality of the selected compounds, the Full Minimization module in D.S. was deployed. This module utilized the CHARMm force field to perform energy minimization on the small molecules. This process resulted in the identification of 141,723 small molecules exhibiting favorable characteristics. These selected molecules were deemed suitable for subsequent docking experiments, ensuring a focused and efficient exploration of potential interactions with the GPD1 protein.

### Docking-based virtual screening

Following the preparation of proteins and small molecules, various modules of D.S. software, including LibDock and CDocker, were employed for docking-based virtual screening. Scoring was conducted based on LibDockScore and -CDOCKER_INTERACTION_ENERGY (-CDIEY) to enhance docking accuracy and efficiency.

#### Docking using LibDock

In the initial docking phase, the faster LibDock module within D.S. software was utilized for screening. This method relies on the properties of points on the protein, categorizing thermal regions into polar and non-polar regions. Ligands with polar atoms preferentially bind to polar thermal regions, while non-polar atoms show an inclination towards non-polar thermal regions. This approach allows for the scoring of different conformations of ligand molecules, aiding in identifying potential binding modes.

#### Docking using CDocker

To further refine the selection of small molecules and enhance screening accuracy, CDocker^21^ was employed to evaluate the remaining set of molecules from the previous step. CDocker utilizes the CDocker algorithm for molecular docking, enabling finer docking. In this process, proteins remain rigid while ligand molecules undergo flexibility. The docking method involves the CHARMm grid format, utilizing high-temperature M.D. principles to randomly generate the ligand’s initial conformation and refine the small molecule’s conformation through lattice-based annealing. The scoring parameter -CDIEY was used to identify better-bound ligands for further investigation.

#### Analyze ligand pose

The ligands selected through molecular docking underwent analysis of their interaction with the receptor protein using the Analyze Ligand Poses module in D.S. This analysis provided a histogram and heat map illustrating the ligand-receptor interactions. The results offer insights into the binding mode of the ligand to the receptor, highlighting reference residues. This information assists in the selection of the desired ligand small molecules for further study.

### Molecular dynamic simulation

M.D. simulations of protein–ligand complexes were conducted utilizing GROMACS2020.6^22,23^. The output files, generated in an aqueous solution through GAUSSIAN09, were converted to fch format files to serve as input files for Multifwn3.8 (dev), facilitating the calculation of RESP atomic charges^24,25^. Small molecules underwent processing with AmberTools18 to create input files for GROMACS. The charges obtained from Multifwn calculations were then incorporated into these files, and protein–ligand complexes were constructed in preparation for kinetic simulations.

The TIP3 water model was employed, and ions were added to maintain the electrical neutrality of the complex system. The system underwent energy minimization with ligand and protein positions constrained. Subsequently, the temperature was set to 298 K, and the pressure mode was configured to Berendsen for system equilibration over 100 ps. The formal simulation involved setting the protein-ligands as a group, restricting the translation and rotation of the center of mass, and independently controlling the protein–ligand group and other material groups to maintain room temperature. The pressure mode was then adjusted to Parrinello-Rahman. Kinetics were executed using a GeForce RTX3060Ti GPU for 100 ns.

The M.D. simulations, spanning 100 ns in the production process, were instrumental in assessing the stability of various small molecules at selected active sites. Trajectory files generated from the simulations were analyzed using VMD (Visual Molecular Dynamics)^26^ for further insights into the dynamic behavior of the protein–ligand complexes.

### Free energy calculations

In this study, gmx_MMPBSA was employed to compute the free energies of the complexes, with subsequent visualization and analysis conducted using gmx_MMPBSA_ana^27^. The gmx_MMPBSA tool facilitates end-state free energy calculations from GROMACS molecular dynamics trajectories using the MM-(PB/GB) S.A. (Molecular Mechanics/Poisson Boltzmann (Generalized Born) Surface Area)^28^. The equation of MM-(PB/GB) S.A. (Molecular Mechanics/Poisson Boltzmann or Generalized Born Surface Area) approach.

The calculation of the binding free energy ($\Delta {G}_{bind}$) for proteins and small molecules in solution is expressed by the equation^28^:

1 $$\Delta {G}_{bind} = \Delta {G}_{complex} - \Delta {G}_{protein} - \Delta {G}_{ligand}$$

The individual components of free energy ($\Delta G$) can be computed as follows:

2 $$\Delta {G}_{bind}= \Delta {E}_{MM}+ \Delta {G}_{solv}-T\Delta S$$

where:

3 $$\Delta {E}_{MM}= \Delta {E}_{bonded}+\left(\Delta {E}_{elec}+ \Delta {E}_{vdW}\right)$$

and

4 $$\Delta {G}_{solv}= \Delta {G}_{polar}+ \Delta {G}_{non-polar}= \Delta {G}_{PB\_GB}+ \Delta {G}_{SA}$$

The MM/PB (G.B.) S.A. methodology is utilized to calculate the difference in binding free energies between the bound and unbound states of molecules in a solvent model or to compare the free energies of different conformations of the same molecule. The binding free energy ($\Delta {E}_{MM}$) in the gas phase is introduced to account for the direct calculation of energy in solution, with the total binding energy divided into the binding free energy of the gas phase and the solvation energy ($\Delta {G}_{solv}$).

$\Delta {E}_{bonded}$ represents the internal energy comprising bond, bond angle, and dihedral angle energies. $\Delta {E}_{elec}$ denotes non-bonded electrostatic energy, while $\Delta {E}_{vdW}$ represents non-bonded van der Waals energy. The term $-T\Delta S$ accounts for conformational entropy changes after ligand binding, typically obtained from normal mode analysis on a set of conformations derived from M.D. simulations^29^. In cases where only the relative binding free energy of similar ligands is required, the change in conformational entropy can often be disregarded.

$\Delta {G}_{solv}$ encompasses the electrostatic solvation energy ($\Delta {G}_{PB\_GB}$) and the nonpolar contribution ($\Delta {G}_{SA}$) between the solute and the continuous solvent. The polar contribution is typically computed using the Poisson Boltzmann (P.B.) or Generalized Born (G.B.) models, while the nonpolar energy is usually estimated using the solvent-accessible surface area (SASA) approach^30^.

## Results and discussion

### Docking-based virtual screening

#### Molecular docking

This study employed two docking procedures for protein structure-based virtual screening. Following an initial series of screenings of compounds in the ZINC database, 141,723 compounds were identified for subsequent molecular docking. Three positive small molecules—Compound 4, Compound 2, and Compound 1—were specifically selected for molecular docking using CDocker, utilizing the -CDIEY value as a reference (Table 1).

**Table 1:** Small molecules as reference and some parameters.

Entry	Ligand	IC_50_ (μM)	CDIEY (kcal/mol)
1	Compound 1	4.1	− 61.5588
2	Compound 2	4.5	− 62.0277
3	Compound 4	11.5	− 64.7938

The primary virtual screening utilized the LibDock program, aiming to retain homologous small molecules within each conformation. This was achieved by limiting the selection to the top 14,000 highest-scoring small molecules based on the scoring function LibDockScore and the diversity of molecular structures. Subsequently, a secondary virtual screening was conducted using the CDocker program, considering the parameters of the positive molecules. To enhance the diversity of small molecules, reduce resulting error, and extend the reference range, a final set of 1,000 small molecules was reserved for detailed analysis, with a threshold set at -CDIEY > 57 kcal/mol.

#### Pose analysis and screening

In this phase, a comprehensive analysis of the complex system involving 1000 small molecules and the GPD1 protein was conducted using D.S.’s ligand pose analysis program. The aim was to determine the contribution of specific residues in binding to small molecules. The involvement of distinct residues in most systems was evaluated, shedding light on their crucial role in the binding process. Table 2 and the Heat Map data highlighted the pivotal residues, including ARG269, LYS204, VAL92, ILE152, LYS296, TRP14, PRO94, and LYS120. These residues played a significant role in multiple systems, influencing the overall system stability.

**Table 2:** Simultaneously Interacting Residues of 1000 compounds.

Rank	Residue 1	Residue 2	Residue 3	Residue 4	Residue 5	Ligand percentage
1	B:TRP14	B:PRO94	B:LYS120	B:LYS204	B:ARG269	5.10%
2	B:PRO94	B:LYS120	B:LYS204	B:ARG269	B:LYS296	3.60%
3	B:VAL92	B:PRO94	B:LYS120	B:LYS204	B:ARG269	3.40%
4	B:PRO94	B:LYS120	B:ILE152	B:LYS204	B:ARG269	3.30%
5	B:TRP14	B:PRO94	B:LYS120	B:ILE152	B:ARG269	3.20%
6	B:PRO94	B:LYS120	B:LYS204	B:ARG269		25.60%
7	B:TRP14	B:PRO94	B:LYS120	B:ARG269		24.10%
8	B:TRP14	B:LYS120	B:LYS204	B:ARG269		19.60%
9	B:TRP14	B:PRO94	B:LYS204	B:ARG269		18.10%
10	B:TRP14	B:PRO94	B:LYS120	B:LYS204		17.10%
11	B:PRO94	B:LYS120	B:ARG269			62.30%
12	B:LYS120	B:LYS204	B:ARG269			48.30%
13	B:PRO94	B:LYS204	B:ARG269			46.90%
14	B:TRP14	B:LYS120	B:ARG269			45.60%
15	B:PRO94	B:LYS120	B:LYS204			45.30%
16	B:PRO94	B:ARG269				76.30%
17	B:LYS120	B:ARG269				75.40%
18	B:PRO94	B:LYS120				68.00%
19	B:LYS204	B:ARG269				56.20%
20	B:TRP14	B:ARG269				54.40%

This table presents twenty sets of data, each consisting of five residues. These residues represent the most common combinations observed in the binding conformational analysis of 1000 molecules. The percentage indicates the frequency of occurrence of each residue combination among all the possible binding configurations. For instance, in rank 1, the five residues are present in 5.1% of all conformations. Notably, in rank 16, two residues, PRO94 and ARG269, are present in 76.3% of conformations, indicating their high prevalence and significance in the binding interactions.

**Figure 4:**
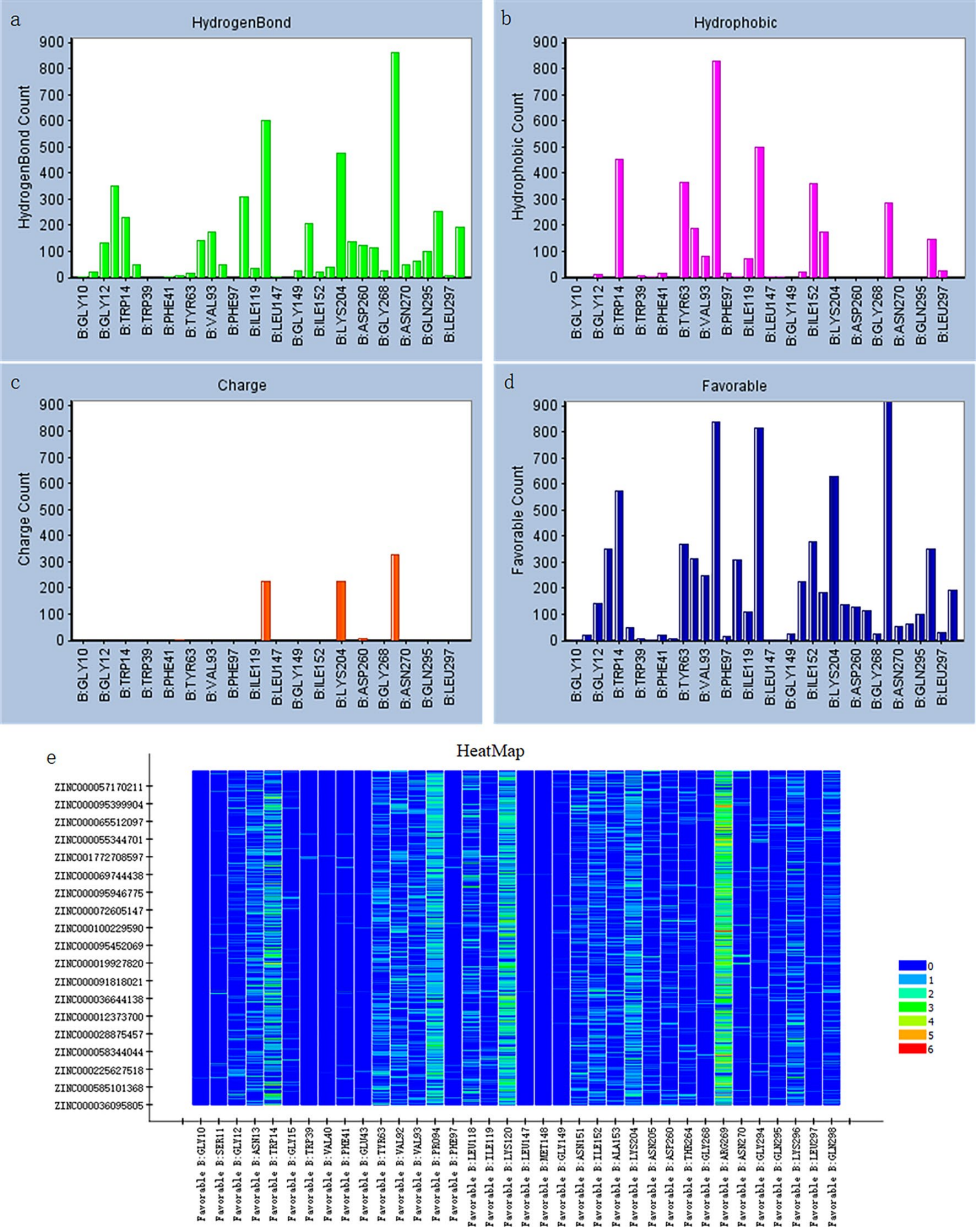
(**a**–**d**) Interaction histogram. (**e**) Heat map of residues with 1000 ligands.

The top three residues facilitating hydrogen bonding or charge interactions were identified as ARG269, LYS204, and LYS120. Meanwhile, the top three residues involved in hydrophobic interactions were PRO94, LYS120, and TRP14. Notably, PRO94 exhibited the widest range of action, whereas TRP14 had a more localized impact (Fig. 4). The variability in the binding patterns of different residues to small molecules provided valuable insights into the GPD1 protein's binding status with ligands. Additionally, it indicated that small molecules showing enhanced binding to the GPD1 protein were likely to involve the mentioned crucial residues. This information serves as a foundation for supporting higher-throughput pharmacophore screening and the identification of potential lead compounds based on specific requirements.

#### Analysis of ligand–protein binding modes using CDOCKER

**Figure 5:**
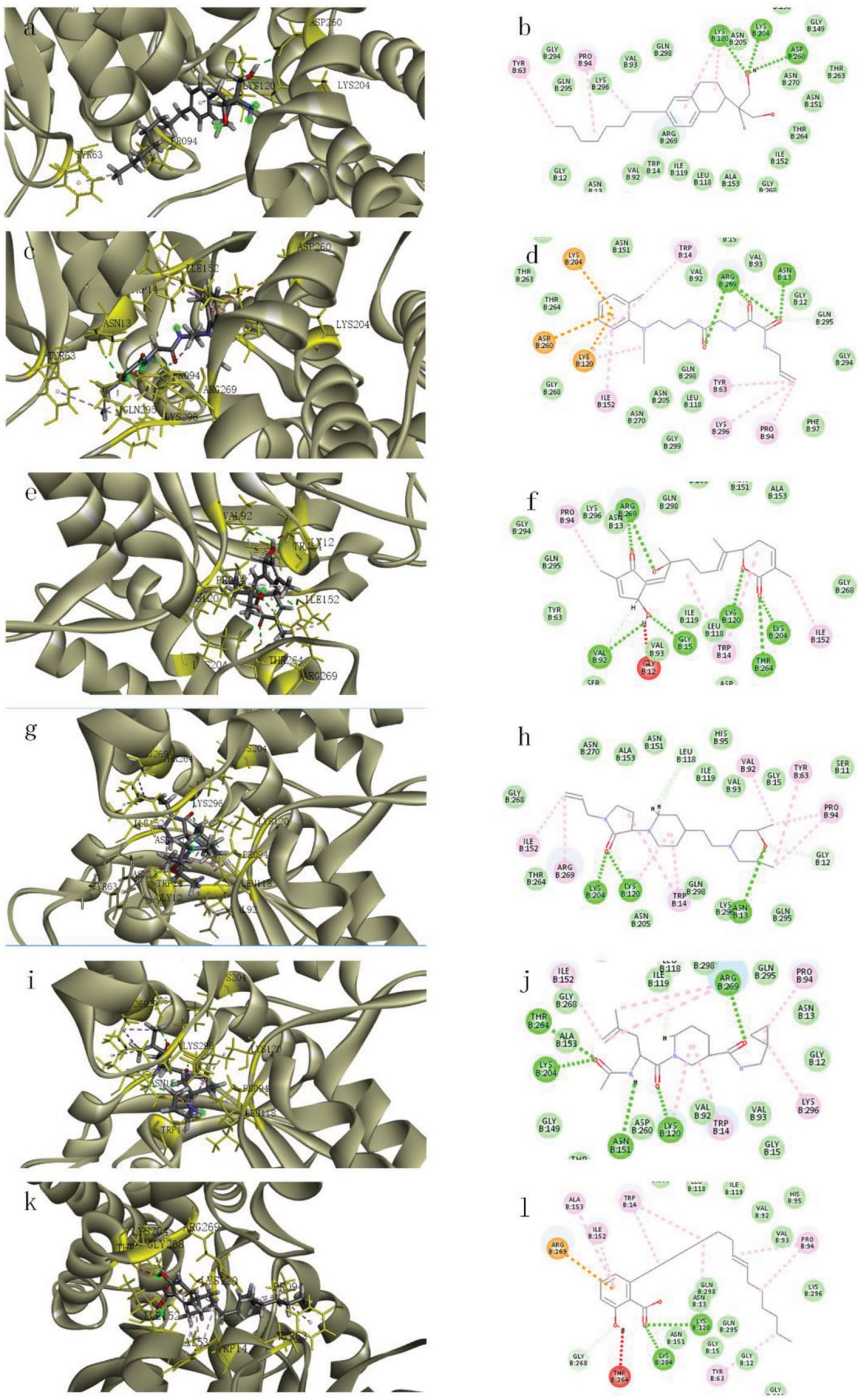
Interaction mode diagram of five molecules. This figure illustrates the presumed binding modes of five selected molecules with the protein. The color scheme distinguishes the ligands (grey sticks) and the receptor (grey ribbon), while different types of interactions are represented by colored dotted lines: green for hydrogen bonds, pink for hydrophobic interactions, orange for Pi-Charge interactions, and red for unfavorable Donor-Donor interactions. (**a**,**b**) Presumed binding mode of ZINC000049780570 with protein. (**c**,**d**) Presumed binding mode of ZINC000058282139 with protein. (**e**,**f**) Presumed binding mode of ZINC000077257642 with protein. (**g**,**h**) Presumed binding mode of ZINC000225627518 with protein. (**i**,**j**) Presumed binding mode of ZINC000585101368 with protein. (**k**,**l**) Presumed binding mode of compound 4 with protein.

Following Pose Analysis and CDOCKER results, five compounds were selected for further examination due to their diverse residue interactions and molecular structures. These ligands exhibited strong binding affinities to the proteins, ranging from − 62.9450 kcal/mol for ZINC000225627518 to − 66.4986 kcal/mol for ZINC000077257642 (Table 3). In these six complex systems, key residues such as LYS120, LYS204, and ARG269 predominantly engaged in hydrogen bonding interactions with oxygen atoms. Notably, in cases like protein binding to ZINC000058282139, where a benzene ring is present at the end of the molecular chain, LYS120 and LYS204 formed charge interactions with the benzene ring. Additionally, residues PRO94 and TPR14 contributed to hydrophobic interactions with the ligands, with PRO94 displaying a broader range of hydrophobic interactions (Fig. 5).

**Table 3:** Five selected molecules and their -CDIEY values.

Entry	ID number	Structure	CDIEY (kcal/mol)
1	ZINC000585101368		− 64.6131
2	ZINC000225627518		− 62.9450
3	ZINC000049780570		− 66.1754
4	ZINC000058282139		− 64.4536
5	ZINC000077257642		− 66.4986

The residues LYS120, LYS204, ARG269, PRO94, and TPR14 played pivotal roles in these binding interactions. While the bonding species of residues align with Pose Analysis, significant heterogeneity exists, as expected.

### Molecular dynamics simulation

**Figure 6:**
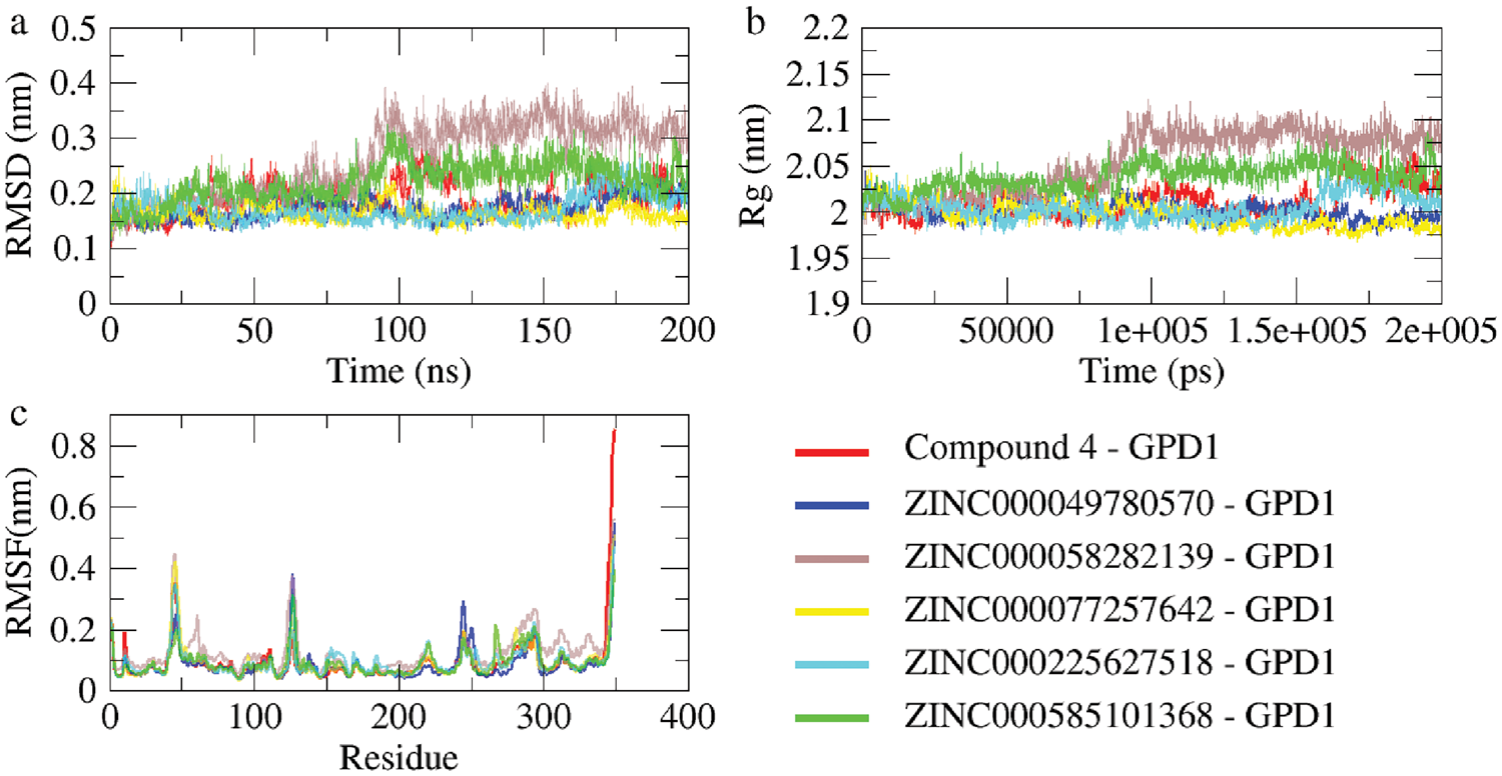
(**a**) RMSD values across the entire simulation duration of protein–ligand complexes. (**b**) The radius of gyration values calculated during M.D. simulations. (**c**) Variation in RMSF of the $\mathrm{C\alpha }$ atoms of key residues within GPD1.

M.D. simulations spanning 200 ns were conducted for five selected ligands exhibiting favorable binding properties alongside the reference small molecule Compound 4. Root mean square deviation (RMSD) was employed to assess the dynamic stability of each system. The average RMSD parameters for Compound 4-GPD1, ZINC000049780570-GPD1, ZINC000058282139-GPD1, ZINC000077257642-GPD1, ZINC000225627518-GPD1, and ZINC000585101368-GPD1 were approximately 0.17, 0.22, 0.23, 0.16, 0.15, and 0.21 nm, respectively (Fig. 6a). Notably, discernible differences were observed in the alteration of the GPD1 structure induced by various small molecules. Compared to Compound 4, ZINC000049780570, ZINC000077257642, and ZINC000225627518 exhibited enhanced systemic stability post-binding, while complexes containing ZINC000058282139 and ZINC000585101368 displayed lower consistency.

The radius of gyration (Rg) serves as a metric for assessing the compactness of protein structures and monitoring changes in the peptide chain's degree of looseness during simulation. Relative to the Compound 4-GPD1 complex, ZINC000049780570-GPD1 displayed similar changes in Rg, while ZINC000077257642-GPD1 and ZINC000225627518-GPD1 exhibited superior behavior. Conversely, the performance of ZINC000585101368-GPD1 was subpar, with ZINC000058282139-GPD1 showcasing progressively enlarged Rg values and a mutation peak at a later stage, indicating its instability (Fig. 6b).

Root Mean Square Fluctuation (RMSF) values serve as crucial indicators of atomic position changes, shedding light on protein flexibility and the extent of motion during M.D. simulations. These values provide insights into residue contributions to the complex system's stability. Calculated from a 10 ns M.D. trajectory with a relatively stable RMSD, the sequence of residues with more stable Rg values is illustrated in Figures 15, 90, 120, 150, 200, and 260. These residues predominantly reside within the protein's active pocket, emphasizing key residues' pivotal role in complex stability (Fig. 6c).

Upon examining the overall graphical trend, it becomes evident that ZINC000077257642, ZINC000225627518, and ZINC000049780570 exhibit significantly restricted movement of residues within the system. This observation underscores the commendable performance of these three small molecules, indicating their potential as robust candidates for further exploration and research. The collective analysis of RMSD, Rg, and RMSF parameters strengthens the evidence supporting the efficacy and research value of ZINC000077257642, ZINC000225627518, and ZINC000049780570 in the examined protein–ligand complexes (Fig. 6c).

### Free energy calculations

#### MM-GBSA binding free energy

To assess the strength of small molecule binding to GPD1 and discern the impact of key residues, the last 50 ns of the M.D. trajectory was extracted for MM-GBSA analysis calculations. The free binding energy of five selected small molecules and the positive reference small molecules to GPD1 was determined (Table 4).

**Table 4:** Binding free energies (kcal/mol) of each complex calculated by the MM-GBSA method.

Complexes	Energy (kcal/mol)						
	${\Delta G}_{vdw}$	${\Delta G}_{ele}$	$\Delta {G}_{PB\_GB}$	$\Delta {G}_{SA}$	${\Delta G}_{gas}$	${\Delta G}_{solv}$	total
Compound 4-GPD1	− 41.17	− 50.15	51.57	− 5.96	− 91.32	45.61	− 45.71
ZINC000049780570-GPD1	− 46.15	− 43.20	61.66	− 6.52	− 89.35	55.14	− 34.21
ZINC000058282139-GPD1	− 12.58	− 4.26	11.68	− 1.79	− 16.84	9.89	− 6.94
ZINC000077257642-GPD1	− 42.13	− 51.41	63.87	− 5.82	− 93.54	58.05	− 35.49
ZINC000225627518-GPD1	− 38.24	− 9.03	23.02	− 5.12	− 47.27	17.90	− 29.37
ZINC000585101368-GPD1	− 19.36	− 6.60	16.32	− 2.63	− 25.97	13.69	− 12.28
Compound 36-GPD1	− 22.54	− 146.27	161.67	− 3.80	− 168.81	157.86	− 10.94
Compound 87-GPD1	− 39.06	− 12.96	25.87	− 4.47	− 52.03	21.41	− 30.62
Compound 95-GPD1	− 40.67	− 1.55	16.45	− 5.28	− 42.22	11.17	− 31.05
Compound 785-GPD1	− 30.24	− 6.46	19.81	− 4.00	− 36.70	15.81	− 20.89
Compound 16-GPD1	− 40.77	− 22.21	33.20	− 5.55	− 62.98	27.64	− 35.34

Total = $${\Delta G}_{gas}$$  + $${\Delta G}_{solv}$$, $${\Delta G}_{gas}$$ = $${\Delta G}_{vdw}$$  + $${\Delta G}_{elec}$$, $${\Delta G}_{solv}$$ = $$\Delta {G}_{PB\_GB}$$  + $$\Delta {G}_{SA}$$.

Table 4 shows that complexes ZINC000058282139-GPD1 and ZINC000585101368-GPD1 exhibit the least binding intensity, aligning with their less favorable RMSD and RMSF values. Conversely, the other three complex systems demonstrate robust adhesive strength comparable to the control. Notably, while the Compound 4-GPD1 complex demonstrates relatively good binding strength, its RMSD and RMSF parameters exhibit less favorable performance compared to ZINC000049780570-GPD1, ZINC000077257642-GPD1, and ZINC000225627518-GPD1. This variance may be attributed to the distinct effects of key residues on the protein. In essence, both the bonding strength and the spatial variability of key residues play pivotal roles in influencing protein stability.

#### Per-residue decomposition analysis

**Figure 7:**
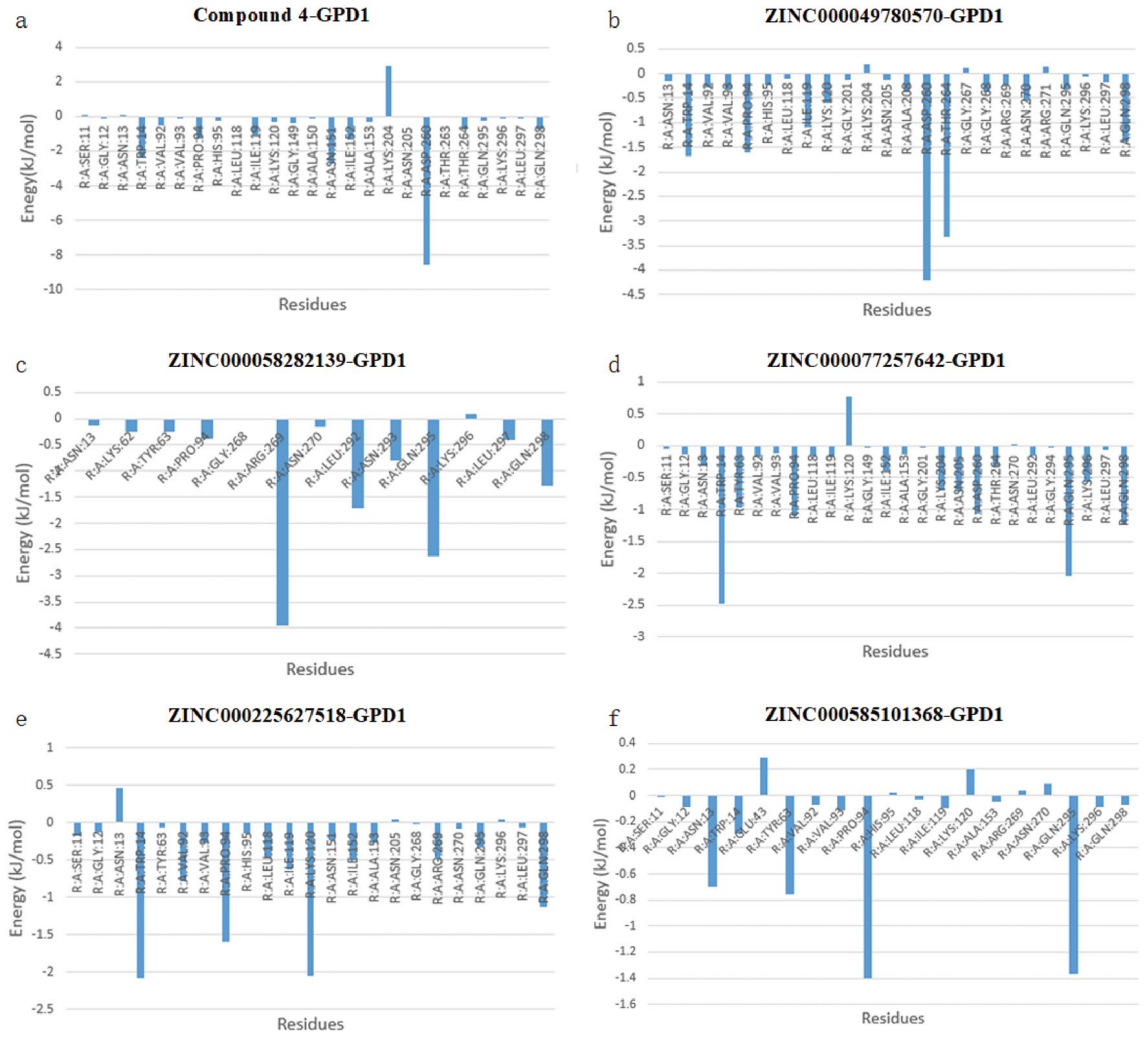
Per-residue decomposition. (**a**) Energy decomposition of complex Compound 4-GPD1. (**b**) Energy decomposition of complex ZINC000049780570-GPD1. (**c**) Energy decomposition of complex ZINC000058282139-GPD1. (**d**) Energy decomposition of complex ZINC000077257642-GPD1. (**e**) Energy decomposition of complex ZINC000225627518-GPD1. (**f**) Energy decomposition of complex ZINC000585101368-GPD1.

**Figure 8:**
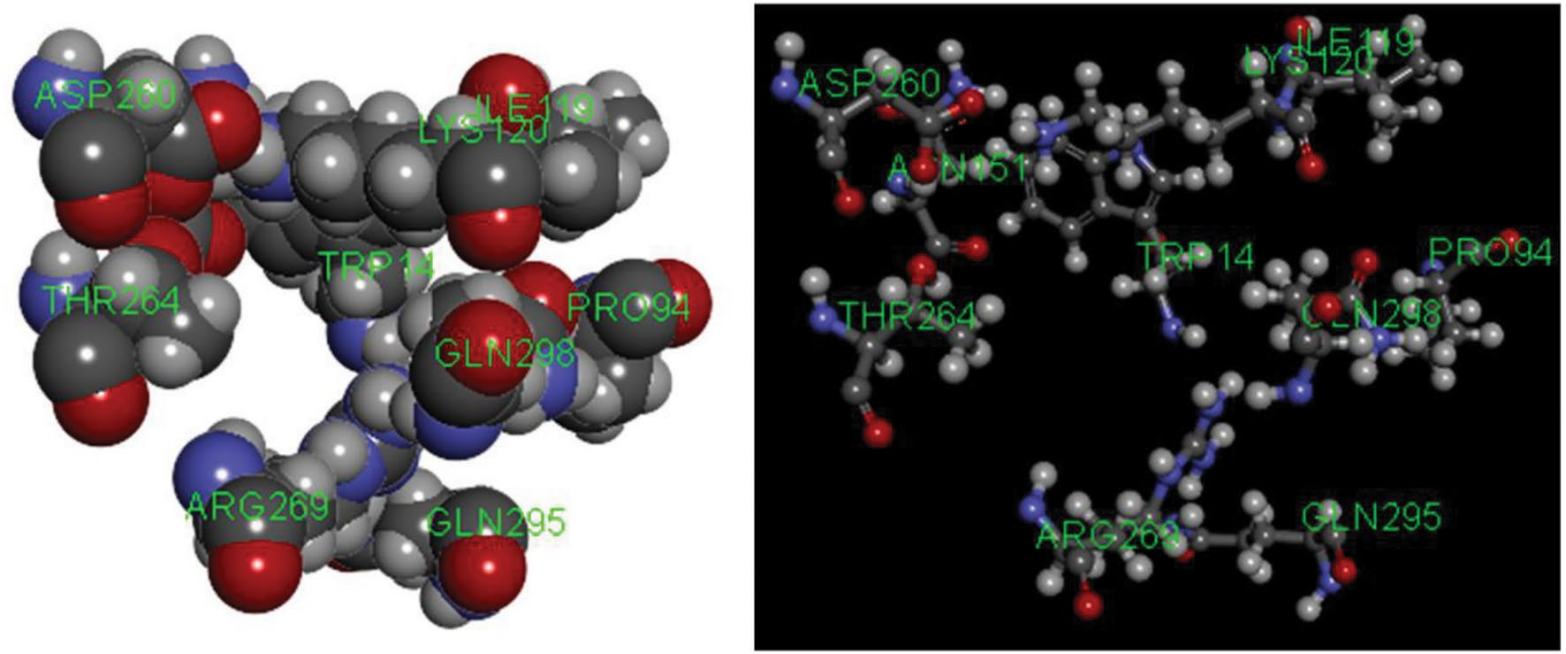
Spatial distribution of some key residues.

The gmx_MMPBSA_ana tool was employed to scrutinize the decomposition energies from the free binding energy calculations, shedding light on residues with binding free energies below -1.0 kJ/mol that exerted substantial control (Fig. 7). Notably, exceptions in complex systems may arise. For instance, despite the high-performing residue ARG269 in ZINC000058282139-GPD1, it did not significantly contribute to system stability, suggesting potential inefficacy. Conversely, the combination of ARG269, LEU292, and GLN295 as key residues demonstrated minimal impact on enhancing system robustness. The complexes Compound 4-GPD1 and ZINC000049780570-GPD1 shared similar key residues, with the latter exhibiting superior stability and a more uniform distribution of residue energy. This observation suggests that a uniformly distributed decomposition energy of residue resembles an equilibrium state, emphasizing better system stability. Conversely, a compact spatial distribution of key residues, as seen in the ZINC000058282139-GPD1 complex, corresponds to less stability. Favorable stability is evident when key residues are spatially distributed sterically or uniformly, as observed in ZINC000049780570-GPD1 and ZINC000225627518-GPD1. The distribution (Fig. 8) and decomposition energy (Fig. 7) of key residues indicate that residues ARG269 and GLN295 may compete with others, influencing overall stability. Complexes containing residues TRP14, PRO94, LYS120, ASN151, THR264, ASP260, GLN298, etc., demonstrate good stability and binding strength, aligning with the Pose Analysis results and emphasizing their utility in analyzing key residues.

### Design of drug-like analogues

Utilizing DeLA-Drug, three molecules—ZINC000049780570, ZINC000077257642, and ZINC000225627518—were subjected to DeLA-Drug calculations to obtain drug-like and synthesizable molecules^31^. Post libdock, CDocker, and ADMET, 556 molecules were acquired. Recognizing that solely relying on software scoring functions in M.D. simulation and subsequent free energy calculations could be computationally wasteful, a step was introduced before M.D. simulation. The Binding Energy module in D.S. software was employed to estimate molecular docking results initially. Subsequently, promising molecules were selected for stability evaluation via M.D. The last 10 ns trajectory was extracted, and 25 frames at intervals were used to calculate the average binding free energy to calculate the average binding free energy to determine their compatibility with the protein.

**Figure 9:**
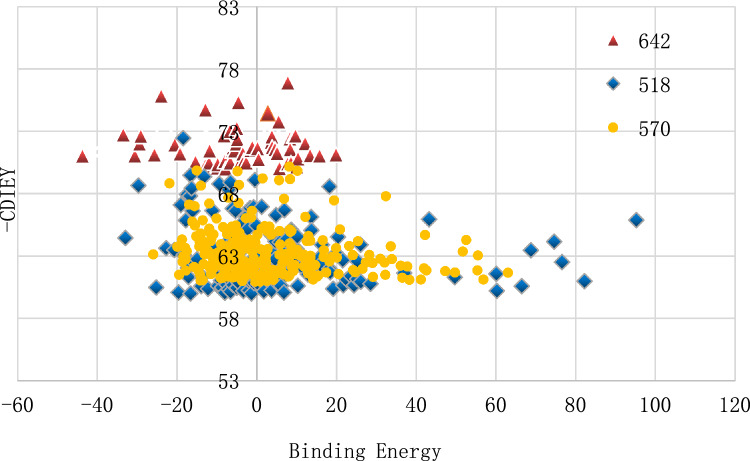
Scatter distribution of small molecules. The red triangle, blue diamond, and yellow circle represent small molecules obtained by the DeLA-Drug calculation of ZINC000077257642, ZINC000225627518, and ZINC000049780570, respectively.

To explore the relationship between molecular docking and binding free energy and optimize results, a scatter diagram for the two parameters was generated (Fig. 9). Significant insights were obtained by observing the oval shapes narrowing from top to bottom and widening from left to right. The trend of decreasing absolute value of binding free energy with increasing -CDIEY values guides the selection of small molecules with better binding performance. This insight is crucial for high-throughput screening and docking results, providing guidance for further screening based on the relationship. However, this approach may pose challenges for teams lacking D.S. software due to limitations in convenient high-throughput calculations for evaluating binding energy.

**Figure 10:**
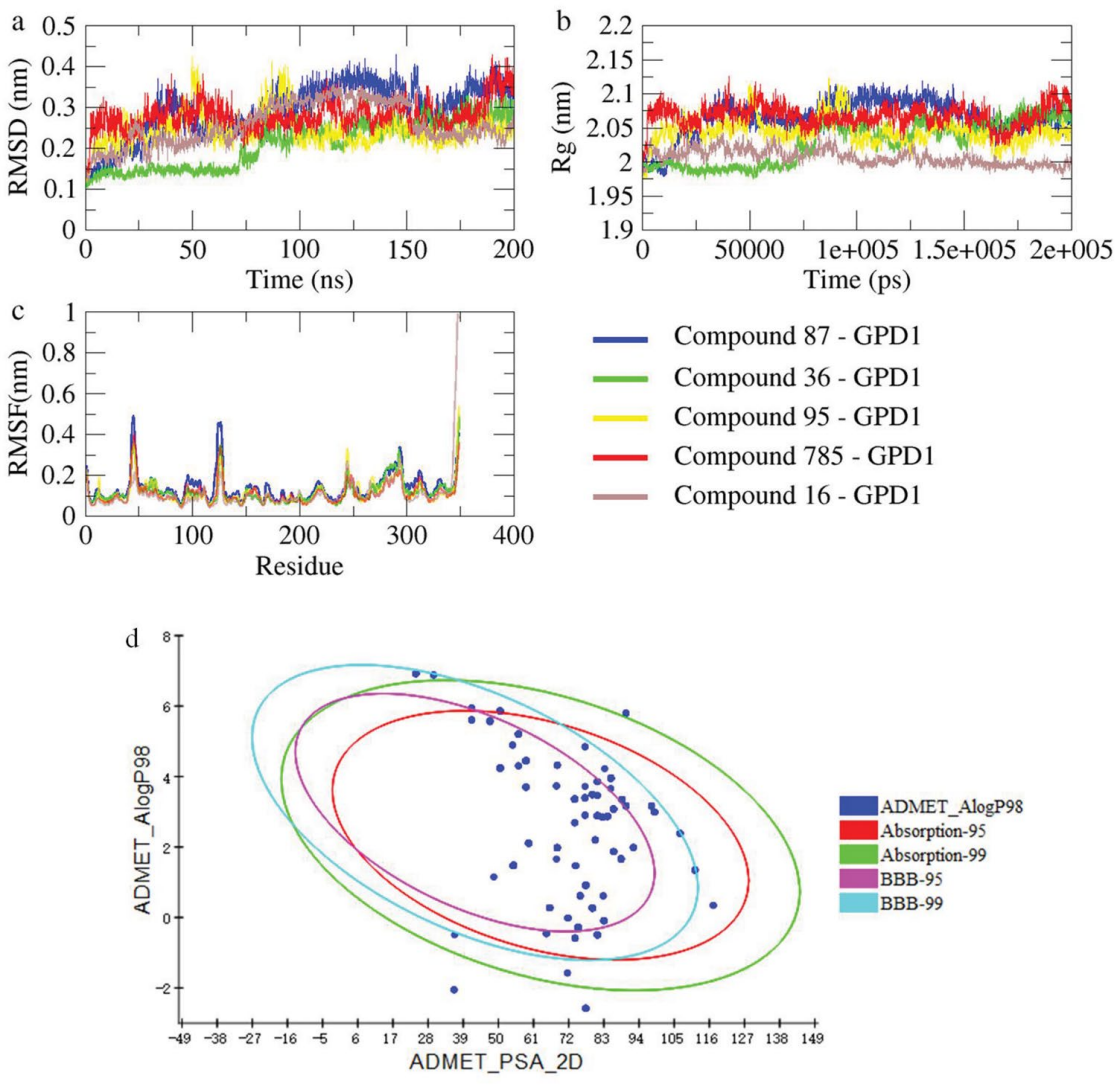
(**a**) RMSD, (**b**) Rg, and (**c**) RMSF parameters of the protein skeleton in the M.D. process. (**d**) Two series of ellipses representing the 95% and 99% confidence intervals of the blood–brain barrier permeability (BBB) model and the 95% and 99% confidence intervals of the human intestinal absorption (HIA) model.

The results (Fig. 10) demonstrate the excellent performance of small molecules designed by DeLA-Drug. This underscores the feasibility of the additional step. Moreover, most generated molecules fall within the 99% confidence interval, providing structural and medicinal reference value. Small molecules selected based on D.S. binding energy showcase commendable stability and binding affinity (Table 5).

**Table 5:** Entry 1, 2, and 3 are selected based on D.S. binding energy. Entry 4 is selected based on -CDIEY. Entry 5 is randomly selected based on the middle value range of -CDIEY.

Entry	Hits	Structure	-CDIEY (kcal/mol)	Binding energy (kcal/mol)
1	Compound16		66.01	− 42.10
2	Compound95		68.83	− 24.00
3	Compound785		71.06	− 28.04
4	Compound87		76.90	− 30.39
5	Compound36		63.09	4.24

## Conclusion

This study conducted a comprehensive exploration, initially winnowing down the screening database through Lipinski, Veber, and ADMET criteria. After this, two distinct molecular docking approaches were employed: rigid docking (Libdock) and semi-flexible docking (CDOCKER). Out of the 1000 small molecules initially selected, five were meticulously chosen for experimental validation, considering conformational diversity and the outcomes of pose analysis. These shortlisted molecules, alongside a reference compound, underwent molecular dynamics simulations. The ensuing binding free energy calculation served as a yardstick for assessing system stability and protein–ligand binding strength.

Results showcased three complexes with superior stability, mirroring the binding strength of Compound 4. Decomposition energy analysis pinpointed residue sequences—TRP14, PRO94, LYS120, ASN151, THR264, ASP260, and GLN298—as pivotal for system stability, emphasizing the importance of their energy and spatial distribution. Notably, findings from Pose Analysis underscored the critical roles of residues such as PRO94, LYS120, LYS204, TRP14, and ASP269 in the complex system. The congruence between Pose Analysis and decomposition energy results enhances their combined reference value.

In the design phase, precision in small molecule selection was augmented by incorporating estimated binding free energy. The outcomes demonstrated commendable stability and binding affinity. This investigation approach and its findings offer valuable insights for future research on GPD1 protein-related studies and the refinement of associated lead compounds. The observed trend relationship between -CDIEY and binding energy suggests potential implications beyond this specific protein complex. If validated in other protein complexes, it may influence the significance of high-throughput molecular screening, potentially making small molecular approximation deformation and designing a mainstream approach. Further studies, particularly leveraging molecular dynamics simulations on the 10 small molecules over 200 ns, promise deeper insights in future research endeavors.

### Supplementary Information

Supplementary Information.

## Data Availability

The datasets used and/or analyzed during the current study are deposited at https://pan.baidu.com/s/1qQICAwop8cbz5j2egE4Ohw, and the extraction code is HAZS.

## Abbreviations

ADMET: Absorption, distribution, metabolism, excretion and toxicity
BTSCs: Brain tumor stem cells
-CDIEY: -CDOCKER_INTERACTION_ENERGY
DHAP: Dihydroxyacetone phosphate
D.S.: Discovery Studio 2019
E-M pathway: Embden-Meyerhof glucose glycolysis pathway
FAD: Flavine adenine dinucleotide
GPD1: Cytosolic glycerol-3-phosphate dehydrogenase 1 (EC 1.1.1.8)
G3P: Glycerol-3-phosphate
GPD2: Mitochondrial glycerol-3-phosphate dehydrogenase 2 (EC 1.1.5.3)
M.D.: Molecular dynamics
RMSD: Root mean square deviation
Rg: Radius of gyration
RMSF: Root mean square fluctuation
